# Dietary trends from 1950 to 2010: a Dutch cookbook analysis

**DOI:** 10.1017/jns.2019.3

**Published:** 2019-02-19

**Authors:** Marjolein E. Buisman, Jochem Jonkman

**Affiliations:** Operations Research & Logistics Group, Wageningen University, PO Box 8130, 6700 EW Wageningen, The Netherlands

**Keywords:** Dietary trends, Recipe analysis, Home cooking, Meal planning, DNFCS, Dutch National Food Consumption Survey

## Abstract

Dietary trends and changing lifestyle patterns have been associated with the increasing occurrence of obesity in the Western world. These dietary trends are commonly studied using longitudinal food consumption surveys. An alternative to studying changes in eating behaviour may be found in recipe analysis of traditional cookbooks. Few such studies exist, however, and it is unclear whether dietary trends over time can be identified this way. The present paper analyses full-meal recipes from a traditional Dutch cookbook between 1950 and 2010. The selected recipes show an increase in energy density. Additionally, the protein weight per kcal increased. In general, the observed trends are similar to those identified by the Dutch National Food Consumption Survey. The analysis therefore suggests that traditional cookbooks can be used as an indicator to identify dietary trends over time, although further studies are necessary to support this hypothesis.

The increasing occurrence of obesity in the Western world has led to studies analysing the changes in dietary patterns of the population over time. A recent report from the United States Department of Agriculture concludes that there was an increase in food intake from all food groups between 1970 and 2010^(^^1^^)^. Similarly, the study of Cohen *et al*.^(^^2^^)^ observes an increase in overall energy intake in the USA, with a percentage-wise decrease of energy from fats, mainly due to an increase in the amount of energy from carbohydrates. In Europe, the overall energy intake and the consumption of meat, fruit, vegetables, nuts and oils increased between 1961 and 2004, while changes in the intake of other food groups differed between Northern, Central and Mediterranean Europe^(^^3^^)^. However, for the specific case of the Netherlands, Hulshof *et al*.^(^^4^^)^ observed a reduction in overall energy intake, while the percentage-wise intake of energy from plant proteins and carbohydrates increased between 1978 and 1997.

As an alternative to studying changing dietary patterns through nutritional surveys, cookbook recipes could provide an indication of the types and quantities of foods consumed at the time of publishing. However, only a few studies are available in the literature. The (retracted) analysis of a US cookbook by Wansink & Payne^(^^5^^)^ found an increase in mean energy content per serving size and an increased average energy density per serving between 1936 and 2006. Similarly, Eidner *et al*.^(^^6^^)^ observed an increased energy content per portion in Danish recipes between 1909 and 2009. Furthermore, an increase was observed within the food group intake of meat, starch products and sauce. The observed trends in both studies are in line with the conclusions on changing dietary patterns mentioned above. This raises the question whether recipes in a Dutch cookbook similarly reflect the observed changes in dietary patterns in the Dutch context.

In this brief report we compare recipes from a traditional Dutch cookbook over the period from 1950 to 2010 based on their nutritional content and we discuss the trends observed in this analysis in relation to those found by the Dutch National Food Consumption Survey (DNFCS).

## Methods

### Cookbook and recipe selection

The Dutch cookbook ‘Margriet Kookboek’ contains basic recipes for daily usage in a Dutch household. According to the publisher, more than 1·2 million copies were sold over the last decades, giving it a high coverage of the 7·86 million Dutch households counted by the Dutch Central Bureau of Statistics^(^^7^^)^. The first edition of this cookbook was published around 1945 (exact date unknown) whereas the latest edition was published in 2010. For this study the edition from 1950 (which is an estimation of publication date; the official date is unknown), and those from 1970, 1989 and 2010 were selected^(^^8^^–^^11^^)^.

Recipes were selected based on their suitability to serve as a full meal according to the standards for a Dutch main course. A typical Dutch dinner consists of a starch source, vegetables and a protein product. In former times, most Dutch dinners contained potatoes, vegetables and meat (if it could be afforded), whereas currently more varied starch and protein sources are used (e.g. pasta, rice)^(^^12^^)^. Although most dinners will contain the three mentioned elements, there are exceptions such as a soup that functions as main course, or a cheese fondue. Recipes that did not describe a main course for dinner, or which according to their description would require additional dishes to form a full meal were excluded from the analysis.

The ingredients of a recipe were classified into several groups (staples, vegetables, protein, oil and fat, others), and a standardised form was used to express the more qualitative descriptions (e.g. one onion) in quantitative terms. For this, an internal cookbook weight reference list was used^(^^9^^,^^11^^)^. For items not covered by these lists, the Dutch Food Composition Database (DFCD)^(^^13^^)^ and the digital food monitoring application of the Netherlands Nutrition Centre^(^^14^^)^ were used. The nutritional content of the selected recipes was calculated based on the DFCD. The form to enter the recipes into a spreadsheet was coded using Visual Basic for Applications and Microsoft Office Excel. An example of the worksheet and the resulting database of recipes are available as Supplementary material.

### Statistical analysis

Data were analysed using the software package SPSS statistics 23 (IBM). Levene statistics showed that data did not have homogeneity of variances. Therefore, comparison between all cookbooks was done with the Kruksal–Wallis test, with a *P* value of 0·05 for the significance level. When a significant difference was found, a *post hoc* analysis was performed with the Mann–Whitney test. For this test, the *P* value has to be adapted based on the number of pairwise comparisons executed. Comparisons with the Mann–Whitney test were performed for 1950–1970, 1970–1989, 1970–2010 and 1989–2010, with the adjusted *P* value of 0·0125 for the significance level. All considered recipes from the 1950 edition still existed unchanged in the 1970 edition, which is an edition extended with new recipes. Due to the high similarity of the recipes in the 1950 and the 1970 edition, the pairwise comparisons for 1950–1989 and 1950–2010 were omitted to reduce the number of required Mann–Whitney tests.

## Results

In total, 187 recipes from the four different cookbooks met the selection criteria described in the previous section and were further analysed. The number of recipes selected per cookbook, the average energy content per portion and the portion weight are presented in Table 1. The analysis of the cookbooks showed no statistically significant difference between the cookbooks of 1950 and 1970, nor between the cookbooks of 1989 and 2010 for any of the macronutrients or energy value of the recipes. An overall decrease of portion weight was observed. However, Table 2 shows that recipe portion weight per 100 kcal (418 kJ) also significantly decreased between the years 1970–1989 and 1970–2010. Similarly, there is a decrease in the quantity of staples in a recipe per 100 kcal between these years, although the quantity of protein ingredients (e.g. meat, cheese) increased per 100 kcal.

**Table 1.** Recipes’ energy content and portion weight, and number of recipes selected from each cookbook (Mean values and standard deviations; numbers)

	1950		1970		1989		2010	
	Mean	sd	Mean	sd	Mean	sd	Mean	sd
Energy (kcal)†	782·9	268·0	723·2	261·4	610·8	211·9	571·8*	192·6
Portion weight (g)	846·6	253·3	761·4	304·6	531·2*	170·8	453·7*	166·2
Number of recipes	26		49		45		67	

* Mean value was significantly different from that for 1970 (*P*<0·0125; Mann–Whitney *post hoc* testing).

† To convert kcal to kJ, multiply by 4·184.

**Table 2.** Portion weight (g) and weight of ingredient groups (g/100 kcal (418 kJ)) for all recipes from each cookbook (Mean values and standard deviations)

	1950		1970		1989		2010	
	Mean	sd	Mean	sd	Mean	sd	Mean	sd
Portion weight (g)	112·11	25·67	109·07	35·97	92·69*	33·86	84·36*	33·98
Staples	43·45	23·24	36·44	25·70	20·99*	16·75	17·42*	16·73
Oil and fat	1·49	1·26	1·65	1·67	1·72	1·03	1·83	2·24
Vegetables	40·72	25·64	42·19	38·90	32·42	23·05	32·00	24·87
Protein foods	8·63	4·87	11·18	7·60	19·10*	14·12	16·44*	8·48

* Mean value was significantly different from that for 1970 (*P*<0·0125; Mann–Whitney *post hoc* testing).

Table 3 shows that the increased quantity of protein ingredients in a recipe observed in Table 2 is reflected in an increase in the total protein weight in a recipe per 100 kcal. More specifically, this increase is related to an increase in the quantity of protein from animal origin. Furthermore, a significant decrease was found in the quantity of fibre and carbohydrates per 100 kcal.

**Table 3.** Weight of macronutrients (g/100 kcal (418 kJ)) for all recipes from each cookbook (Mean values and standard deviations)

	1950		1970		1989		2010	
	Mean	sd	Mean	sd	Mean	sd	Mean	sd
Total protein	3·93	1·17	4·27	1·41	5·99*	2·63	5·70*	2·13
Vegetable protein	2·15	0·98	1·92	1·04	1·50	0·70	1·80	0·90
Animal protein	1·78	1·20	2·35	1·69	4·48*	2·94	3·90*	2·54
Total fat	3·61	1·27	4·07	1·59	4·41	1·48	4·43	1·56
Total carbohydrate	11·75	2·88	10·44	3·67	8·27*	4·05	8·50*	3·62
Total mono- and disaccharides	1·40	1·21	1·64	1·25	1·44	1·18	1·64	1·70
Total fibre	2·32	1·21	2·02	1·24	1·37*	0·75	1·35*	0·85

* Mean value was significantly different from that for 1970 (*P*<0·0125; Mann–Whitney *post hoc* testing).

Regarding meat, by far the most recipes contained pork (eighty-seven out of 187), followed by beef (forty-one out of 187), while thirty-eight recipes did not contain any meat or fish. Looking at animal-based ingredients in more detail, the recipes in the cookbooks of 1950 and 1970 contain ingredients such as lard, while these are not found any more in the recipes from the 1989 and 2010 cookbooks. Additionally, the later cookbooks contain more recipes using easy-to-cook meat, such as chicken. While none of the selected recipes from the 1950 cookbook contained poultry, 25 % of the recipes in the 2010 cookbook did. Later recipes also contained ingredients which were traditionally not used in Dutch cooking, such as olives.

## Discussion

The observed overall decrease of portion weight per recipe can be explained by a decrease in the weight of staple foods and vegetables used. The decrease of these generally bulky ingredients also explains the observed reduction in fibre content. Additionally, the decrease of staple foods and vegetables leads to a relative increase of protein ingredients, which are more energy dense. This is also observed by the decreased portion weight per 100 kcal of food. Hence, the reduced portion weight does not lead to a similar reduction of the energy intake per meal.

Given the similarities between the cookbooks of the years 1950 and 1970, the observed differences between the years 1970–1989 and 1970–2010 are expected to exist between 1950–1989 and 1950–2010 as well.

Our study shows a decrease in energy per recipe over the years, which is in line with the DNFCS report of Hulshof *et al*.^(^^4^^)^, but contrary to studies from other countries which found an increase in energy intake^(^^1^^,^^2^^)^. At the level of ingredient groups, our results are in line with the DNFCS. However, although a percentage-wise increase in the energy intake of protein was observed, the cookbook analysis suggests this is related to an increased contribution of animal protein, whereas Hulshof *et al*.^(^^4^^)^ observed an increase in plant-based protein. The increased contribution in the newer cookbooks of easy-to-cook animal protein corresponds to the shift in practice away from red meat^(^^12^^)^. The total meat consumption has continued to decline slightly in recent years^(^^15^^)^, which may be reflected by an increased number of non-meat or low-meat recipes in future editions of the cookbook.

A decrease was observed in the contribution of carbohydrates and fibres to the overall energy intake, where the DNFCS shows an increase in the contribution of carbohydrates^(^^4^^)^. Carbohydrates make up a large part of the energy consumption in-between meals, which nowadays accounts for about 30 % of the daily energy intake, equalling the carbohydrate consumption at dinner^(^^16^^)^. Hence, the analysis of main meals was not able to detect this trend, showing a limitation of not taking into account the entire diet.

In the Dutch diet, food items such as potatoes, vegetables, legumes, meat, condiments and sauces are predominantly eaten during dinner^(^^16^^)^. Trends in the consumption of these food items are therefore more likely to be spotted in the recipe analysis. Although the Dutch are suggested to be conservative in their eating behaviour^(^^12^^,^^17^^)^, an increasing quantity of food seems to be consumed in-between the typical meals^(^^17^^)^. However, this trend was not significant yet for most of the time period covered in this study^(^^12^^)^.

Despite these limitations, the general trends observed in the cookbook analysis correspond to the trends identified in the DNFCS. Previous recipe analysis studies also observe a similarity between trends identified in recipes and trends identified in food consumption surveys^(^^6^^,^^18^^)^. Hence, we conclude that this type of traditional cookbooks is likely to follow actual changes in dietary patterns. It may therefore be possible to use the historic developments in traditional recipes as an indicator of the actual changing dietary patterns over time. This is useful for those cases in which there are no detailed data available from food consumption surveys, although more study is required to further support this observation.

Not all cookbooks will be suitable for such analyses, and the characteristics of suitable cookbooks require further investigation. Additionally, it is not clear how well recipes reflect home cooking practices, as consumers will often deviate from described recipes, or only use them for inspiration. Another limitation to the comparison of recipes throughout time is the possibility of variations in measures, and the interpretation of recipe instructions. The quantities of ingredients lost and wasted, the nutritional content, processing, etc. may differ throughout the years. These variations could affect the results of the analysis.

The results of this cookbook analysis raise the interest in studying other cookbooks and comparing them with results from dietary surveys. An extension of these analyses to the level of micronutrients would also be of interest. Moreover, as suggested by Eidner *et al*.^(^^6^^)^, the relationship between recipes in cookbooks and home cooking practices can be investigated. Additional insights can be obtained from a further investigation of meal elements which combine into a meal, of combinations of meals for a daily consumption pattern, and of multi-day menu planning. This would aid the field of diet modelling, and could for instance be used by cookbook publishers to provide an accompanying web application which suggests recipes that improve an overall nutrient score (for example, using the Nutrient Rich Foods Index as developed by Drewnowski^(^^19^^)^ and Fulgoni *et al*.^(^^20^^)^) based on the meals selected by the consumer on the previous days.

## Supplementary material

For supplementary material accompanying this paper visit https://doi.org/10.1017/jns.2019.3. click here to view supplementary material
